# Factors associated with non-initiation of osteoporosis pharmacotherapy after hip fracture: analysis of claims data in Japan

**DOI:** 10.1007/s11657-023-01314-x

**Published:** 2023-07-21

**Authors:** Tomoko Fujii, Takahiro Mori, Jun Komiyama, Naoaki Kuroda, Nanako Tamiya

**Affiliations:** 1 Health Services Research and Development Center, University of Tsukuba, 1-1-1 Tenno-dai, Tsukuba, Ibaraki, 305-8575 Japan; 2 Faculty of Physical Education, Kokushikan University, Tokyo, Japan; 3 Department of Orthopaedic Surgery, Faculty of Medicine, The University of Tokyo, Tokyo, Japan; 4 Department of General Medicine, International University of Health and Welfare Narita Hospital, Chiba, Japan; 5 Department of Health Services Research, Graduate School of Comprehensive Human Sciences, University of Tsukuba, Ibaraki, Japan; 6 Department of Health Services Research, Institute of Medicine, University of Tsukuba, Ibaraki, Japan; 7 Health Department, Tsukuba City, Ibaraki, Japan; 8 Department of Community Mental Health & Law, National Institute of Mental Health, National Center of Neurology and Psychiatry, Tokyo, Japan

**Keywords:** Care gap, Fragility hip fracture, Osteoporosis, Pharmacotherapy

## Abstract

***Summary*:**

In an analysis of claims data from a city in Japan, male patients and patients with dementia were less likely to receive osteoporosis pharmacotherapy after hip fracture. Treatment initiation rate has improved between 2014 and 2017.

**Purpose:**

Older adults with recent hip fractures are at a high risk of recurrent fractures. However, the post-fracture care gap has been reported globally. This study examines factors associated with pharmacotherapy non-initiation within 1 year after hip surgery.

**Methods:**

Using medical and long-term care (LTC) claims, and LTC needs certification data in Tsukuba City, Japan, we identified individuals aged 65 years or older who had hip fractures with subsequent surgical procedures between October 1, 2014, and December 31, 2017. Patient (age, sex, dementia, and comorbidities) and health service–related characteristics (fiscal year, type of hospital, number of hospital beds, and admission to recovery phase rehabilitation wards) were examined. The association of these factors with non-pharmacotherapy for osteoporosis within 1 year after hip fracture using multivariable logistic models was analyzed.

**Results:**

We identified 275 patients with hip fractures who did not receive pharmacotherapy pre-fracture. Forty percent of them received pharmacotherapy within 1 year of post-fracture. Male sex (odds ratio (OR) = 4.49 [2.14–9.44]) and dementia (OR = 1.90 [1.03–3.52]) were associated with no pharmacotherapy, whereas later fiscal year (OR = 0.64 [0.48–0.87]) and admission to rehabilitation wards (OR = 0.25 [0.14–0.46]) were associated with pharmacotherapy initiation within 1 year of post-fracture. Comorbidities were not associated with the initiation of pharmacotherapy.

**Conclusion:**

Pharmacotherapy for osteoporosis was less likely to be initiated after a hip fracture in male patients and patients with dementia. These patients should be considered for pharmacotherapy because they are at high risk of recurrent fractures.

**Supplementary Information:**

The online version contains supplementary material available at 10.1007/s11657-023-01314-x.

## Introduction

Fragility hip fractures pose a substantial burden on both patients and society. Hip fracture results in disability, reduced quality of life (QOL) [1, 2], and increased mortality [3, 4]. There is a heavy economic burden associated with fragility hip fractures, and the total medical cost for hip fractures was estimated to be USD $2.99 billion per year in Japan [5]. In addition, the risk of subsequent hip fracture increases after the initial hip fracture [6, 7]. Therefore, pharmacotherapy for osteoporosis is crucial for preventing recurrent fractures and reducing the burden on patients and society.

Evidence for the efficacy of osteoporosis pharmacotherapy has been established. Reports indicate that bisphosphonates can prevent secondary hip fractures and reduce mortality [8]. However, the osteoporosis care gap has been reported globally, with reported treatment rates after hip fractures of < 40% [9–12]. In particular, the treatment initiation rate after hip fracture is low [12–14]. Some patient and healthcare provider factors for non-treatment for osteoporosis after fragility fracture include men, chronic kidney disease, patient perception, and clinical judgment [15–17].

To fill the osteoporosis care gap, the fracture liaison service (FLS) was introduced, which provides routine assessment and necessary treatment to patients with a recent fracture. Improvements in outcomes such as bone mineral density (BMD) testing, treatment initiation, treatment adherence, subsequent fracture, and mortality have been reported [18–20]. In Japan, certification of the Osteoporosis Liaison Service (OLS) Coordinator by the Japan Osteoporosis Society was started in 2015 to disseminate the liaison service for osteoporosis treatment [21]. However, patient- and health service–related factors affecting treatment initiation for osteoporosis after hip fracture and improvement in treatment initiation rate in Japan are not fully known.

The purpose of this study was to exploratorily examine the factors associated with the initiation of pharmacotherapy for osteoporosis in patients with hip fractures and to assess whether the initiation rate has changed since 2015, using medical and long-term care (LTC) claims data as well as LTC needs certification data in Japan.

## Methods

### Data sources

The present study utilized medical and pharmacy claims data, LTC claims data, and LTC needs certification data between April 1, 2014, and March 31, 2019, in Tsukuba City, Japan. The city is located in the suburbs of Tokyo, Japan. It had a population of approximately 220,000, and 18% of them were aged 65 years or older in 2014 [22]. The medical and pharmacy claims data included data from the Late Elder’s Health Insurance (public medical insurance for all individuals aged ≥ 75 years and those aged 65–74 years with certified disability) and National Health Insurance (public medical insurance for unemployed or self-employed people and retirees aged < 75 years). The database comprised two health insurance datasets covering 89% of citizens aged ≥ 65 years as of October 2018 [23]. In Japan, most of the population has some form of medical insurance, and a small percentage of adults aged 65 years or older are covered by Employees’ Health Insurance or public assistance. Our database did not include these older adults. All data were anonymized with unique identification numbers, which helped merge the medical claims and LTC data.

### Study population

We identified patients aged 65 years or older who were newly diagnosed with hip fractures and subsequently underwent a surgical procedure between October 1, 2014, and December 31, 2017, using disease codes for hip fractures (femoral neck and trochanteric fractures) and procedure codes (open surgery for femur fracture, bipolar hip arthroplasty, or total hip replacement) (*n* = 561). The index date was defined as the date of the first recorded surgical procedure for hip fractures. In Japan, 95% of patients with hip fractures receive surgical treatment, and the median wait time before surgery is 3 days [24]. In the case of multiple hip fractures during the study period, the first hip fracture of the patient was treated as an index hip fracture. We only included patients who were continuously enrolled for medical insurance in this city for at least 6 months prior to and at least 15 months after a month of the index date (*n* = 428). Withdrawal due to death was noted for 114 patients. If the patients were admitted to hospital beds or facilities under the comprehensive payment system for medical expenses (recovery phase rehabilitation ward, community comprehensive care ward, long-term care bed, and geriatric health services facilities), drug prescription data may not have been available. Therefore, we excluded patients with hip fractures who were in these hospital beds or facilities during the 3 months before (*n* = 22) or 12 months consecutively (*n* = 39) after the index month using medical and LTC claims data. Older patients remain admitted if they need rehabilitation or need to prepare for returning home or transferring to care facilities after their condition has stabilized. In addition, these patients remain admitted if they require long-term medical care after the acute medical care, nursing care, and support (due to disabilities) under a physician’s supervision until they return home. Additionally, we excluded patients who had disease codes for bone metastasis during the 15 months following the index month (*n* = 3) to focus on fragility hip fracture cases. None of the patients had disease codes for Paget’s disease.

### Osteoporosis medications

**Fig. 1:**
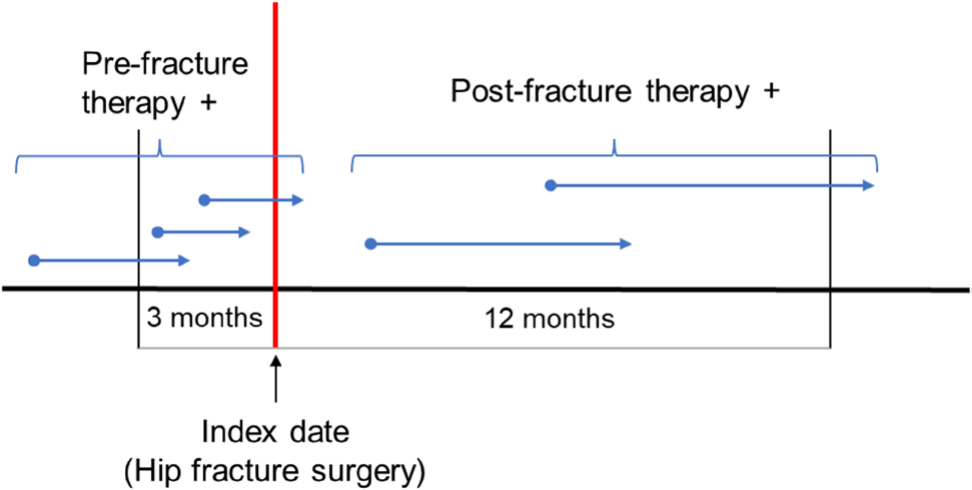
Pharmacotherapy for osteoporosis pre- and post-fracture

The study outcome was pharmacotherapy for osteoporosis, defined as the occurrence of at least one medical or pharmacy claims for osteoporosis pharmacotherapy within 12 months post-fracture (Fig. 1). We determined the prescription of drugs (oral and parenteral) with approved indications for osteoporosis treatment in Japan. These included bisphosphonates (alendronate, risedronate, minodronate, ibandronate, etidronic acid, and zoledronic acid), selective estrogen receptor modulators (raloxifene and bazedoxifene acetate), receptor activator of nuclear factor kappa-B ligand antibodies (denosumab), parathyroid hormone (both daily and weekly), active vitamin D_3_ single-agent (eldecalcitol), and others (alfacalcidol, calcitriol, menatetrenone, ipriflabon, calcium aspartate, calcium phosphate, and elcatonin).

Osteoporosis pharmacotherapy within 90 days prior to the index date was determined based on 6-month prescription data before the index date. The last drug-available day was estimated based on the date of the prescription and the number of days of supply according to the types of osteoporosis drugs in each prescription.

### Assessment of co-variables

The types of hospital (national or public, university, and other hospitals) where the patients underwent hip fracture surgery, the number of beds in each hospital, and admission to the recovery phase rehabilitation ward within three months after the index month were identified from medical claims data. Other fractures within 1 year of the index hip fracture were identified using diagnostic codes. As a proxy for general comorbidity, the Charlson comorbidity index (CCI) score was calculated using the International Statistical Classification of Diseases and Related Health Problems (ICD-10) codes in the three months preceding the index months with the algorithms by Quan et al., which has been previously used in medical and long-term care claims data in Japan [25–27].

Anti-dementia drugs (donepezil, rivastigmine, galantamine, and memantine), proton pump inhibitors (PPI), non-steroidal anti-inflammatory drugs (NSAIDs), and steroids prescribed within 90 days before the index date were ascertained using medication codes. Polypharmacy was defined as the median number of prescribed drug components within 3 months prior to the index month being ≥ 5.

LTC needs certification determined the eligibility and care need level (7 levels: support 1, support 2, care 1, care 2, care 3, care 4, and care 5) of individuals aged ≥ 65 years or those aged 40–64 years with disability due to specific diseases. The certification is based on an assessment of physical and cognitive functions as well as the opinion of the patients’ primary physicians. LTC certification data includes the graded activities of daily living related to dementia noted by a primary physician with eight grades and one missing category: Grade 0, I (almost independent daily living is possible despite having some type of dementia), IIa, IIb, IIIa, IIIb, IV, and M (marked psychiatric symptoms requiring expert management). In Japan, most people aged ≥ 65 years with dementia applied for LTC needs certification [28]. For LTC service recipients, a cutoff of grade I or greater had 83% sensitivity and 92% specificity with the gold standard diagnosis made by neuropsychiatrists [28]. Hence, we defined patients with probable dementia as those who had ≥ grade I in the latest LTC needs certification before the index date, had diagnosis codes of dementia within 3 months prior to the index months, or were prescribed anti-dementia drugs 90 days preceding the index date [29].

### Analysis

Initially, the percentages of participants with and without osteoporosis pharmacotherapy before hip fracture but who received pharmacotherapy within 1 year after hip surgery were calculated.

To focus on pharmacotherapy initiation, only patients with hip fracture without osteoporosis pharmacotherapy within 90 days before the index date were included. Patient- and health service–related characteristics at baseline, admission to the recovery phase rehabilitation ward within 3 months, and other fractures within a year of initial hip fracture were compared between patients with and without osteoporosis pharmacotherapy within 1 year of post-fracture. This was done using *t* tests for continuous variables and chi-square tests or Fisher’s exact tests for categorical variables. The four types of hospitals (university hospitals, national hospitals, public hospitals, and other hospitals) were categorized into two types: university, national or public hospitals, and other hospitals. The number of hospital beds was categorized into one of three ranges: 100–199, 200–499, and ≥ 500. The CCI was categorized into one of three groups: 0, 1–2, and ≥ 3. The LTC care needs levels were categorized into one of four groups: without certification, support 1 and 2, care 1–3, and care 4 and 5. We also considered two kinds of dementia variables: the use of anti-dementia medication, and probable dementia based on LTC needs certification, disease codes, or anti-dementia medication use before hip fracture. Pharmacotherapy initiation rates post-fracture were calculated by fiscal year.

The association between the characteristics and pharmacotherapy for osteoporosis within 1-year post-fracture was examined exploratorily using multiple logistic regression models. The outcome was no pharmacotherapy within 1 year after the index date. Age at surgery and sex were included in the models, regardless of statistical significance. Owing to the limited number of outcomes and avoiding saturated models, the independent variables were chosen based on their clinical relevance, the results of the chi-square tests, and better model fit based on Akaike’s information criterion (AIC). For example, hospital type and number of hospital beds were related; however, only the number of hospital beds was included in the model because of the better model fit than hospital type. The fiscal year of hip surgery was treated as a linear variable after verifying that the overall results did not change if it was treated as a categorical variable. In the sensitivity analysis, instead of dementia, the LTC care needs level was included in the logistic regression model. Adjusted odds ratios (ORs) and corresponding 95% confidence intervals (CIs) were estimated. All analyses were conducted using SAS 9.3 (SAS Institute Inc. Carny, NC), and an alpha level of 0.05 was defined as statistically significant.

### Ethical approval

All procedures performed in this study were in accordance with the ethical standards of the institutional and/or national research committee and with the 1964 Helsinki declaration and its later amendments or comparable ethical standards. For this type of study, formal consent is not required. This study was approved by the Medical Ethics Committee of the University of Tsukuba (approval number 1445-10).

## Results

**Fig. 2:**
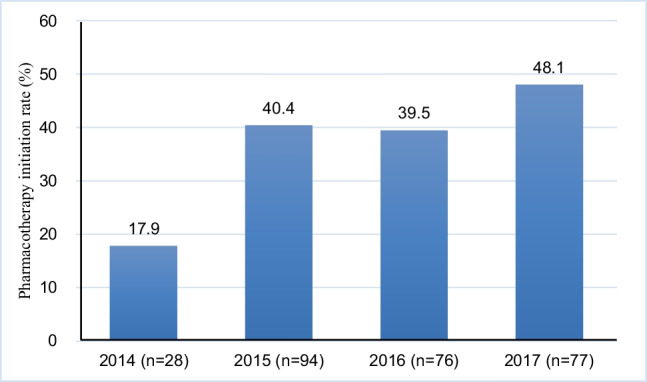
Osteoporosis pharmacotherapy initiation rate by year (*n* = 275)

We identified 364 patients with hip fractures (males: 21.4%). Among these, 89 (24.5%) had osteoporosis pharmacotherapy prior to hip fracture, and 275 (75.6%) did not. In patients with pharmacotherapy pre-fracture, almost all patients (96.6%) received osteoporosis pharmacotherapy within 1 year of post-fracture. However, in patients without pharmacotherapy pre-fracture, only 110 (40.0%) patients received osteoporosis pharmacotherapy within 1 year of post-fracture. The rates of pharmacotherapy initiation by fiscal year are shown in Fig. 2. The initiation rate was higher in later years.

The mean age of the 275 patients without pharmacotherapy for osteoporosis pre-fracture was 83.9 (standard deviation = 7.6), and 25.8% (*n* = 71) were males. The characteristics of patients are listed in Table 1. Among these 275 participants, those who did not receive pharmacotherapy post-fracture were more likely to be males, underwent hip surgery in hospitals other than university, national, or public hospitals, or were treated in hospitals with < 500 beds. They were also more likely to have probable dementia or be prescribed anti-dementia drugs before the hip fracture, require a high care need level, not be admitted to the recovery phase rehabilitation ward within 3 months, and have no other fractures within 1 year of the hip fracture. No association was found between polypharmacy and osteoporosis pharmacotherapy after hip fracture.

**Table 1:** Characteristics of hip fracture patients according to osteoporosis pharmacotherapy dispensed within 1 year after hip fracture (*n*=275)

	Without pharmacotherapy	With pharmacotherapy	*p* value ^a^
	(*n*=165)	(*n*=110)	
Mean age at hip fracture (SD)	84.2 (8.2)	83.5 (6.7)	0.424
Age at hip fracture (%)			0.348
65–74	23 (13.9)	12 (10.9)	
75–79	22 (13.3)	20 (18.2)	
80–84	28 (17.0)	26 (23.6)	
85–89	47 (28.5)	30 (27.3)	
≥90	45 (27.3)	22 (20.0)	
Sex (%)			0.003
Male	53 (32.1)	18 (16.4)	
Female	112 (67.9)	92 (83.6)	
Types of surgery (%)			Not tested
Osteosynthesis	101 (61.2)	71 (64.6)	
Total hip arthroplasty	0 (0)	1 (0.9)	
Bipolar hip arthroplasty	64 (38.8)	38 (34.6)	
Fiscal year (%)			0.050
2014	23 (13.9)	5 (4.6)	
2015	56 (33.9)	38 (34.6)	
2016	46 (27.9)	30 (27.3)	
2017	40 (24.2)	37 (33.6)	
Hospital type (%)			0.017
University, national or public hospital	6 (3.6)	12 (10.9)	
Other	159 (96.4)	98 (89.1)	
Hospital beds number			0.008
100–199	29 (17.6)	14 (12.7)	
200–499	133 (80.6)	85 (77.3)	
≥ 500	3 (1.8)	11 (10.0)	
CCI			0.682
0	56 (33.9)	43 (39.1)	
1, 2	64 (38.8)	39 (35.5)	
≥3	45 (27.3)	28 (25.5)	
Myocardial infarction	8 (4.9)	5 (4.6)	0.908
Congestive heart failure	32 (19.4)	21 (19.1)	0.950
Peripheral vascular disease	11 (6.7)	15 (13.6)	0.053
Cerebrovasucular disease	47 (28.5)	31 (28.2)	0.956
Dementia	49 (29.7)	16 (14.6)	0.004
Chronic pulmonary disease	32 (19.4)	23 (20.9)	0.758
Rheumatic disease	4 (2.4)	1 (0.9)	0.651
Peptic ulcer disease	43 (26.1)	24 (21.8)	0.422
Mild liver disease	21 (12.7)	17 (15.5)	0.521
Diabetes without complication	40 (24.2)	31 (28.2)	0.465
Diabetes with complication	8 (4.9)	12 (10.9)	0.058
Hemiplegia or paraplegia	3 (1.8)	1 (0.9)	0.652
Renal disease	11 (6.7)	6 (5.5)	0.683
Malignancy	15 (9.1)	10 (9.1)	1.000
Moderate or severe liver disease	1 (0.6)	0 (0)	1.000
Metastatic solid tumor	1 (0.6)	0 (0)	1.000
AIDS/HIV	0 (0)	0 (0)	
Probable dementia ^b^	97 (58.8)	44 (40.0)	0.002
Anti-dementia drugs	37 (22.4)	11 (10.0)	0.008
PPI	51 (30.9)	35 (31.8)	0.873
NSAIDs	42 (25.5)	26 (23.6)	0.732
Steroid	2 (1.2)	2 (1.8)	1.000
Polypharmacy (≥5) ^c^	88 (53.3)	49 (44.6)	0.153
Care needs level			0.0002
No certification, independent	65 (39.4)	59 (53.6)	
Support 1,2	6 (3.6)	8 (7.3)	
Care 1–3	64 (38.8)	41 (37.3)	
Care 4,5	30 (18.2)	2 (1.8)	
Admission to rehabilitation ward	50 (30.3)	66 (60.0)	<.0001
Other fractures within 1 year	13 (7.9)	19 (17.3)	0.017

*SD* Standard deviation, *CCI* Charlson comorbidity index, *PPI* proton pump inhibitors, *NSAIDs* non-steroidal anti-inflammatory drugs

^a^*p* value from t-test for continuous variables, chi-square test or Fisher’s exact test for categorical variables

^b^Probable dementia defined as ≥ grade I in activities in daily living related to dementia in long-term care (LTC) needs certification, diagnoses code for dementia, or anti-dementia drugs use before hip fracture

^c^Polypharmacy defined as median of the number of prescribed drug components within three months prior to the index month ≥ 5

The results of the multivariable logistic regression analysis are presented in Table 2. The final logistic model included age at surgery, sex, probable dementia, CCI, fiscal year of hip surgery, number of hospital beds, and admission to the recovery-phase rehabilitation ward within 3 months (model 1). Sex, probable dementia, fiscal year, and no admission to the recovery phase rehabilitation wards were significantly associated with no osteoporosis pharmacotherapy within 1 year after the hip fracture. Male patients were four times more likely to have no therapy (OR = 4.49 [2.14–9.44]). Patients with probable dementia were almost twice as likely to not receive osteoporosis pharmacotherapy after hip fracture (OR = 1.90 [1.03–3.52]). Later fiscal years were negatively associated with no therapy (OR = 0.64 [0.48–0.87]). Patients admitted to recovery phase rehabilitation wards post-fracture were more likely to receive osteoporosis pharmacotherapy (OR = 0.25 [0.14–0.46]). CCI was not associated with pharmacotherapy after hip fractures. When anti-dementia medication usage before hip fracture instead of probable dementia was included in the model, the overall results did not change (model 2). Anti-dementia drug usage was significantly associated with no pharmacotherapy (OR = 2.66 [1.12–6.30]), and patients treated in hospitals with ≥ 500 beds were more likely to receive pharmacotherapy for osteoporosis than those treated in hospitals with 200–499 beds (OR = 0.21 [0.05–0.95]). In the analysis using the LTC care needs level, instead of dementia, a high LTC care needs level (level 4 or 5) was significantly associated with non-therapy (OR = 12.54 [2.54–61.79], Supplementary table).

**Table 2:** Multivariable logistic analysis for non-pharmacotherapy for osteoporosis within 1 year after hip fracture (*n*=275)

Model 1	Odds ratio [95% CI]^a^	Type III *p* value	Model 2	Odds ratio [95% CI]^a^	Type III *p* value
Age		0.100	Age		0.031
65–74	1.72 [0.62, 4.75]		65-74	1.41 [0.53, 3.74]	
75–79	0.80 [0.32, 1.95]		75-79	0.71 [0.30, 1.71]	
80–84	0.68 [0.30, 1.53]		80-84	0.60 [0.27, 1.34]	
85–89	Reference		85-89	Reference	
≥90	2.01 [0.91, 4.44]		≥90	2.28 [1.03, 5.06]	
Sex		<.0001	Sex		<.0001
Male	4.49 [2.14, 9.44]		Men	4.86 [2.29, 10.28]	
Female	Reference		Women	Reference	
Probable dementia ^b^		0.042	Anti-dementia medication		0.026
No	Reference		No	Reference	
Yes	1.90 [1.03, 3.52]		Yes	2.66 [1.12, 6.30]	
CCI		0.489	CCI		0.614
0	Reference		0	Reference	
1, 2	1.50 [0.77, 2.95]		1, 2	1.37 [0.69, 2.72]	
≥3	1.18 [0.57, 2.43]		≥3	1.04 [0.49, 2.18]	
Fiscal year	0.64 [0.48, 0.87]	0.004	Fiscal year	0.66 [0.49, 0.90]	0.007
Hospital beds number		0.033	Hospital beds number		0.020
100–199	1.89 [0.85, 4.19]		100-199	2.03 [0.91, 4.51]	
200–499	Reference		200-499	Reference	
≥ 500	0.24 [0.06, 1.01]		≥ 500	0.21 [0.05, 0.95]	
Admission to rehabilitation ward		<.0001	Admission to rehabilitation ward		<.0001
No	Reference		No	Reference	
Yes	0.25 [0.14, 0.46]		Yes	0.26 [0.15, 0.48]	

*CI* confidence interval, *CCI* Charlson comorbidity index

^a^Odds ratio and corresponding 95% confidence intervals were adjusted for other variables

^b^Probable dementia defined as ≥ grade I in activities in daily living related to dementia in long term care (LTC) needs certification, diagnoses code for dementia, or anti-dementia drugs use before hip fracture

## Discussion

Among hip fracture patients without pharmacotherapy for osteoporosis pre-fracture, only 40% received osteoporosis pharmacotherapy within 1 year of hip fracture, whereas 96.6% of patients with pharmacotherapy pre-fracture received osteoporosis pharmacotherapy post-fracture. Among older adults without pharmacotherapy pre-fracture, male patients, those with dementia, and those who were not admitted to the recovery phase rehabilitation ward after hip fracture were associated with no osteoporosis pharmacotherapy initiation. Patients who had hip fractures in the later fiscal years were more likely to receive pharmacotherapy after a hip fracture.

In our study, the overall rate of pharmacotherapy within 1 year after hip fracture was 53.9% in older adults aged ≥ 65 years and 40.0% in those without pharmacotherapy pre-fracture. The overall rate was higher than the recent report of 31.6% in Japan, which included men and women who were ≥ 50 years and diagnosed with hip fractures between 2013 and 2018 [12]. In that study, the rate of pharmacotherapy in those without pharmacotherapy before fracture was 19% based on their table, which was lower than that in the current study. However, a direct comparison was difficult to interpret because their study included younger patients and did not include some drugs (calcitriol, ipriflabon, calcium aspartate, calcium phosphate, and elcatonin) which were included in our study, using the National Database of Health Insurance Claims. In addition, we excluded patients receiving continuous care in hospital beds or care facilities after hip fracture where drug prescription may not appear in claims. Nonetheless, the initiation rate for pharmacotherapy is still suboptimal considering that fragility hip fracture is a criterion for osteoporosis pharmacotherapy initiation regardless of BMD in the current Japanese guidelines [30].

Dementia was associated with non-pharmacotherapy initiation after hip fracture, whereas the burden of comorbid diseases represented by the CCI was not. Oyamada et al. reported the reasons for non-treatment during hospitalization among hip fracture patients receiving initial pharmacotherapy from the perspective of physicians. The reasons include physicians’ clinical judgment and dementia [17]. Although the reasons for the non-treatment of patients with initial therapy were examined, dementia may influence physicians’ decision to prescribe pharmacotherapy for older adults with hip fracture. Based on administrative insurance claims data in the USA, Solomon et al. also reported that dementia was associated with a lack of osteoporosis medication use within 12 months after discharge in patients with hip fractures [9]. The possible factors affecting physicians’ prescription decisions may include patients’ understanding or perception of osteoporosis therapy, as well as the issue of medication adherence in older adults with dementia. Beaton et al. reported that the perceived need of patients was associated with the initiation of osteoporosis pharmacotherapy in patients aged ≥ 50 years who sustained a fragility fracture in Canada [16]. Misconceptions about the disease and lack of perceived benefit of therapy were factors associated with lower medication adherence [31]. Psychiatric conditions, such as depression are associated with poor medication adherence among patients with osteoporosis [31]. García-Sempere et al. reported that dementia was associated with non-adherence following hip fracture in patients aged ≥ 65 years [32]. Physicians may believe that older adults with dementia face difficulty adhering to pharmacotherapy, or that the assessment of adherence by caregivers in older adults may be difficult. However, cognitive impairment is a risk factor for falls which result in hip fractures [33]. Thus, pharmacotherapy for recurrent fracture prevention is beneficial for older adults with dementia.

Other characteristics were associated with pharmacotherapy for osteoporosis after hip fracture. Male patients were over four times less likely to receive pharmacotherapy after a hip fracture than female patients. Lower treatment rates for men were also reported in the recent studies [12, 15]. The risk of subsequent hip fracture is also high in men as in older women [7, 34]. In addition, excess mortality after hip fracture is high in men [4, 35]. Therefore, older men with hip fractures should receive pharmacotherapy to prevent recurrent fractures.

Patients admitted to recovery phase rehabilitation wards within three months after hip fracture were more likely to receive pharmacotherapy within 1 year. It is possible that physicians were more likely to prescribe osteoporosis drugs to patients who were at higher risk of falls and needed rehabilitation after surgery compared to those who did not. In a recent Japanese report, a low Barthel index, which is a common score for activities of daily living, was associated with non-pharmacotherapy [15]. In our sensitivity analysis using LTC care needs levels instead of dementia, admission to the recovery phase rehabilitation wards was still significantly associated with higher pharmacotherapy initiation, adjusting for LTC care needs levels. Over 60% of the patients not admitted to a recovery phase rehabilitation ward were either in their own homes or some form of housing for elderly persons 3 months after the index months. In addition, among the patients not admitted to a recovery phase rehabilitation ward, there were more patients in intensive care homes run by local government or non-profit organizations, which provided long-term care for older adults who required consistent care before the hip fracture and returned after the hip fracture. Fourteen percent were in hospital beds other than those in recovery phase rehabilitation wards. It is possible that patients who were admitted to the rehabilitation wards were more likely to have access to the OLS team or BMD testing during hospitalization than those who left the initial hospital for their houses or other care facilities after surgery; however, we could not ascertain, based on claims data, whether the FLS had adequate access to the patient and their health-care provider in each hospital’s recovery phase rehabilitation wards.

In our study, a later fiscal year was associated with a higher rate of initiation of osteoporosis pharmacotherapy after hip fracture. Similar results were reported in Hokkaido in Japan [36]. The fracture liaison service is a secondary prevention program designed to provide routine assessment and necessary treatment to patients with recent fractures. The improvement of BMD testing, treatment initiation, treatment adherence, and cost-effectiveness has been reported [18–20, 37, 38]. In Japan, OLS includes FLS, and certification of the OLS Coordinator began in 2015 [21]. Patients in our study underwent hip surgery between October 2014 and December 2017. Improvement in the treatment initiation rate is multifactorial. For example, yearly zoledronic acid hydrate was marketed in November 2016 in Japan, which may have influenced physicians’ prescription behavior. In addition, among the overall patients included in the present study, in 2014 only patients who had hip fracture surgery between October 1 and December 31 were included. Seasonal differences regarding patients’ characteristics and medical facilities’ circumstances might have influenced the osteoporosis pharmacotherapy initiation rate in 2014. Therefore, this result should be interpreted with caution. FLS was not covered by medical insurance and did not appear in medical claims during that period. Therefore, direct causal relationship cannot be known. Nonetheless, the educational certification courses for OLS coordinators may have positively impacted the initiation of osteoporosis treatment in Japan.

Older adults who underwent hip fracture surgery in larger hospitals were more likely to receive pharmacotherapy after hip fracture. Possible reasons may include a lack of BMD testing and liaison teams in smaller hospitals; however, based on claims data, we could not determine whether the FLS were active in each hospital. Upon examining claims data, Sugiyama et al. found that process quality measures of diabetes care, such as proportions of patients receiving retinopathy examination, were better in medical institutions with a higher number of beds and institutions with certification as an educational institution by the Japan Diabetes Society [39]. Martin et al. reported in their meta-analysis that interventions targeting health systems as well as healthcare professionals were effective in improving the prescription of osteoporosis therapy [40]. The introduction of a multidisciplinary OLS team in small hospitals or building an OLS network with neighboring hospitals might be useful in improving pharmacotherapy initiation for osteoporosis. However, we did not explore the medical facilities in which these older adults received care after discharge from the initial hospital. Further studies are needed to assess the influence of the characteristics of medical facilities on treatment initiation.

The strength of this study lies in the combined medical claims data, LTC claims data, and LTC needs certification data for this analysis. Hence, we considered the disability levels of hip fracture patients, but excluded patients who continuously stayed in hospitals or care facilities where drug prescription data may not be reported, which has not been highlighted in the previous studies using medical claims data. Our findings of dementia and admission to recovery phase rehabilitation wards as factors associated with pharmacotherapy initiation build on the previous literature. We identified hip fracture patients using both diagnostic and procedure codes, which improved the specificity of diagnosis, although some hip fracture patients without a surgical procedure were not included in our study.

Nonetheless, this study has some limitations. Since we only considered osteoporosis pharmacotherapy for 3 months before hip fracture based on 6-month medical claims data due to data availability, some patients might have received pharmacotherapy earlier. Thus, some misclassifications regarding osteoporosis pharmacotherapy before hip fractures are possible. Additionally, we could not determine whether these older patients used the prescribed medication because we used medical claims data.

In conclusion, pharmacotherapy for osteoporosis was less likely to be initiated after hip fracture in male patients and patients with dementia, although the treatment initiation rate was better in later years. These patients should be considered for pharmacotherapy for osteoporosis because they are at a high risk of falls and recurrent fractures.

### Supplementary information

ESM 1 (DOCX 19 kb)
