# Supplementary material for: Factors associated with non-initiation of osteoporosis pharmacotherapy after hip fracture: analysis of claims data in Japan
Source: Arch Osteoporos. 2023 Jul 21;18(1):103. doi: 10.1007/s11657-023-01314-x (PMC10361872; doi:10.1007/s11657-023-01314-x)
Supplement: Supplementary file 1 — (DOCX 19 kb) [file 11657_2023_1314_MOESM1_ESM.docx]

**Supplementary table:** Multivariable logistic analysis for non-pharmacotherapy for osteoporosis within 1 year after hip fracture (n=275)

|  | Odds ratio [95% CI] ^a^ | Type III p-value |
| --- | --- | --- |
| Age |  | 0.063 |
| 65-79 | 1.30 [0.64, 2.62] |  |
| 80-89 | Reference |  |
| ≥90 | 2.48 [1.16, 5.29] |  |
| Sex |  | <.0001 |
| Male | 4.47 [2.15, 9.28] |  |
| Female | Reference |  |
| LTC care needs level |  | 0.014 |
| No certification, independent | Reference |  |
| Support 1,2 | 0.69 [0.19, 2.47] |  |
| Care 1-3 | 1.34 [0.70, 2.58] |  |
| Care 4,5 | 12.54 [2.54, 61.79] |  |
| CCI |  | 0.457 |
| 0 | Reference |  |
| 1, 2 | 1.53 [0.77, 3.02] |  |
| ≥3 | 1.11 [0.53, 2.31] |  |
| Fiscal year | 0.64 [0.47, 0.87] | 0.004 |
| Hospital beds number |  | 0.027 |
| 100-199 | 1.73 [0.76, 3.91] |  |
| 200-499 | Reference |  |
| ≥ 500 | 0.20 [0.05, 0.84] |  |
| Admission to rehabilitation ward |  | <.0001 |
| No | Reference |  |
| Yes | 0.28 [0.15, 0.52] |  |
| CI, confidence interval; LTC, long-term care; CCI, Charlson comorbidity index | | |
| ^a^ Odds ratio and corresponding 95% confidence intervals were adjusted for other variables | | |
